# Unequal Burdens: Irritable Bowel Syndrome in Sexual and Gender Minority Communities vs Cisgender Heterosexual Individuals

**DOI:** 10.14309/ctg.0000000000000883

**Published:** 2025-07-07

**Authors:** Sara Alejandra Reyes-Diaz, Bryan Adrian Priego-Parra, Héctor Ricardo Ordaz-Alvarez, Emma Lorena Núñez-Jiménez, Claudia Leticia Dorantes-Nava, Fátima Higuera-de la Tijera, Mercedes Amieva-Balmori, Christopher Velez, José María Remes-Troche

**Affiliations:** 1 Departamento de Fisiología Digestiva y Motilidad Gastrointestinal, Instituto de Investigaciones Médico-Biológicas, Universidad Veracruzana, Veracruz, Mexico;; 2 Centro de Investigaciones Biomédicas, Universidad Veracruzana, Veracruz, Mexico;; 3 Departamento de Gastroenterología, Hospital General de México “Dr Eduardo Liceaga, Mexico City, Mexico”;; 4 Center for Neurointestinal Health, Division of Gastroenterology, Massachusetts General Hospital, Harvard Medical School.

**Keywords:** LGBTQIA+, irritable bowel syndrome, anxiety, sexual and gender minority, psychosocial factors

## Abstract

**INTRODUCTION:**

Irritable bowel syndrome (IBS) is a disorder of gut-brain interaction that negatively impacts quality of life. Given the significant health disparities faced by the sexual and gender minority (SGM) communities, it is essential to explore IBS within the context of sexual orientation and gender identity (SOGI). This study aimed to compare the severity of gastrointestinal and psychological symptoms between cisgender heterosexual and SGM individuals with IBS.

**METHODS:**

This cross-sectional study recruited 718 participants, with 60.7% being women and a median age of 22. Of these, 542 (75.5%) identified as cisgender heterosexuals, and 176 (24.5%) identified as SGM. Participants, including both patients with IBS and healthy controls (HCs), completed a 60-item electronic survey addressing SOGI, the Rome IV IBS criteria, the Hospital Anxiety and Depression Scale, and the Irritable Bowel Syndrome Severity Scale (IBS-SSS). Statistical analyses included the Student *t* test, Wilcoxon rank-sum test, Kruskal-Wallis test, and Pearson or Spearman correlations.

**RESULTS:**

SGM individuals with IBS reported significantly higher IBS-SSS scores (*P* = 0.032) and anxiety levels (*P* = 0.032) than their cisgender heterosexual counterparts. In addition, the prevalence of lesbian women was higher in the IBS group compared with healthy controls (*P* = 0.041). Cisgender heterosexual participants were more likely to report mild IBS symptoms compared with LGBTQIA+ participants (*P* = 0.025).

**DISCUSSION:**

SGM individuals with IBS experience more severe symptoms and greater psychological distress compared with cisgender heterosexuals. These findings underscore the need to consider SOGI in health care to ensure that management strategies for IBS are inclusive and effectively address the unique needs of all individuals.

## Graphical abstract

**Figure:**
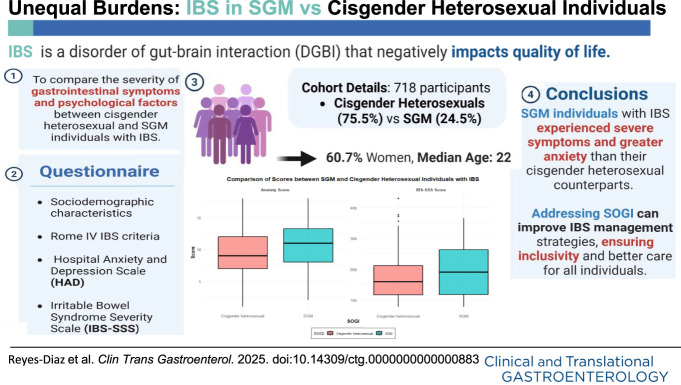

## INTRODUCTION

Irritable bowel syndrome (IBS) is a disorder of gut-brain interaction (DGBI) characterized by chronic abdominal pain and altered bowel habits that decrease the quality of life of those affected. It is often accompanied by high rates of psychological comorbidity as well as other visceral and somatic pain-related symptoms (1). Several neuropsychological factors negatively affect the quality of life in individuals with IBS including high levels of anxiety and depression, chronic stress, emotional dysregulation, and psychosocial stressors such as social anxiety and fear of stigma (2).

Sexual and gender minority (SGM) individuals, commonly referred to as lesbian, gay, bisexual, transgender, queer, intersex, asexual, and others (LGBTQIA+), face unique stressors, including increased psychological burdens, discrimination, and social stigma, compared with their cisgender counterparts. As a result, they experience higher rates of mental health conditions (3). These distinct stressors can intensify the neuropsychological effects of IBS contributing to a more pronounced decline in quality of life. Understanding IBS within the broader context of sexual orientation and gender identity (SOGI) is essential, especially given the significant health disparities faced by SGM individuals, disparities that are often overlooked in healthcare settings (4).

The World Health Organization (WHO) has emphasized that the root causes of health inequities are to be found in the social, economic, and political mechanisms. Despite this, the effects of social determinants, environmental and structural health contexts in marginalized communities have been investigated in only a few studies (5). Although most patients are open to discuss their SOGI and perceive these inquiries as significant for their health-related outcomes (6), these crucial data often remain unrecorded (7). The lack of SGM representation in research studies highlights a significant gap in understanding DGBI's impact on diverse populations.

Recent evidence indicates that SGM individuals exhibit notable neurobiological differences in gray matter volume across the brain, particularly within the central executive, salience, and default mode networks (8). Interestingly, similar alterations in brain structure have been observed in individuals with IBS (9). These neurobiological changes have been linked to visceral hypersensitivity and hyperalgesia (10–12). Taken together, this convergence points to a potential shared neurobiological pathway underlying chronic psychosocial stress, commonly experienced by SGM populations—and the pathophysiology of IBS.

Biological and functional differences between men and women affect gastrointestinal function and pain perception. Although IBS is more commonly diagnosed in women, the influence of self-identified gender and sexual orientation on gastrointestinal symptoms and psychological distress is not well understood (13). Therefore, this study aimed to compare the severity of gastrointestinal and psychological symptoms in individuals with IBS, specifically between cisgender heterosexual individuals and those identifying as SGM.

## MATERIAL AND METHODS

### Study design and participants

In this observational, cross-sectional, and analytical study, we compared patients diagnosed with IBS against healthy controls (HCs) across various states in Mexico. Participants were recruited consecutively from February 2023 to April 2024 through an electronic survey, which was widely disseminated by posters at our institution and on social media.

Although the survey was open to the general public, most responses came from students (71.7%), faculty, and staff affiliated with the University associated with our Research Institute. During the recruitment period, approximately 2,500 individuals were potentially exposed to the invitation to participate. A total of 826 responses were recorded, suggesting an estimated survey response rate of 33.04%. This reflects the reach and engagement within the primary recruitment setting.

### Inclusion and exclusion criteria

Inclusion criteria for all study participants included signing an informed consent form, being between 18 and 79 years of age, being of Mexican nationality, residing in Mexico, and completing all items on the applied questionnaires. Exclusion criteria encompassed illiteracy, non-Spanish speakers, psychiatric or psychological pathologies, any diagnosed organic disease that better explains IBS-type symptoms, and current medication use. According to diagnostic algorithms recommended by Mexican health authorities, given the low diagnostic yield of serologic/stool-based testing and the expense of endoscopic evaluation, those patients not meeting criteria for such evaluation and meeting criteria for IBS were designated as having IBS.

Individuals with IBS were eligible if they met the Rome IV Criteria for IBS and exhibited no alarm signs, such as anemia, weight loss, or fever (14). HCs were defined as those without gastrointestinal symptoms, history of any diagnosed disease, and/or consumption of drugs.

### Data collection

Data collection involved a 60-item electronic survey, which gathered information on sociodemographic characteristics (sex, age, body mass index, academic degree, marital status, medical history, and medication usage), sexual orientation, gender identity, and gastrointestinal and psychological symptoms. To inquire about sexual orientation, participants were asked: Which of the following sexual orientations best represents how you think of yourself? The options provided were heterosexual, homosexual, bisexual, demisexual, and/or asexual, each accompanied by a brief description to aid individuals who may be unfamiliar with these terms. In addition, participants were allowed to write other sexual orientation(s) if the provided options did not represent them. We implemented the same measures to inquire about gender identity, offering the options of cisgender, transgender, nonbinary, and/or genderqueer/other.

### Survey scoring

A certified gastroenterologist performed the IBS diagnosis according to the Rome IV criteria. To measure the severity of IBS symptoms the Irritable Bowel Syndrome Symptom Severity Scale (IBS-SSS) was used. It comprises 5 items that assess the frequency and intensity of abdominal pain and distension, an individual's satisfaction with their bowel movements, and the impact on their quality of life. Scores are reported as a discrete variable (0–500) and categorize symptom severity as follows: none (<75), mild (75–174), moderate (175–300), and severe (>300) (15,16).

The Hospital Anxiety and Depression Scale (HADS) was used to assess levels of anxiety and depression. This questionnaire consists of 14 items divided into 2 subscales: the anxiety subscale (HADS-A) with 7 items, and the depression subscale (HADS-D) with another 7 items. The scale provides both continuous and categorical interpretations. Each item is scored on a four-point scale, from 0 to 3, based on the frequency or intensity of the symptom experienced in the past 7 days. The total score on each subscale ranges from 0 to 21, with a higher score indicating a greater presence of anxiety or depressive symptoms. A cutoff point of 8 has been established as an indicator of clinically significant anxiety or depression (17,18).

### Statistical analysis

Numerical variables were reported using measures of central tendency (means, SDs, medians, and interquartile ranges), whereas categorical variables were expressed as frequencies and percentages. Data distribution was assessed using the Kolmogorov-Smirnov test, and homoscedasticity was examined using the Levene test. For comparisons between groups, the Student *t* test or Wilcoxon rank-sum test was used for numerical variables, depending on the data distribution. Multiple comparisons were conducted using the Kruskal-Wallis test. The χ^2^ test or Fisher exact test was used as appropriate for categorical variables. Correlations between variables were assessed using the Pearson or Spearman test, with significance set at *P* < 0.05. Logistic regression models were constructed at a 95% confidence level. The Nagelkerke pseudo-R^2^ was calculated to assess the proportion of variance explained by the model, and the Hosmer–Lemeshow test was used to evaluate the goodness of fit. Data analysis was performed in R Studio version 4.4.3 and SPSS V. 25 software.

### Ethical considerations

The study protocol was meticulously reviewed and approved by the Ethical and Scientific Committee of the Institute of Medical-Biological Research at Universidad Veracruzana (approval number: IMB-007-2022). The conduct of the research adhered to the ethical standards outlined in the Declaration of Helsinki and complied with federal law for the protection of personal data in the possession of individuals. In line with these standards, all data collected were deidentified to ensure participant confidentiality and stored securely with restricted access.

## RESULTS

**Figure 1.**
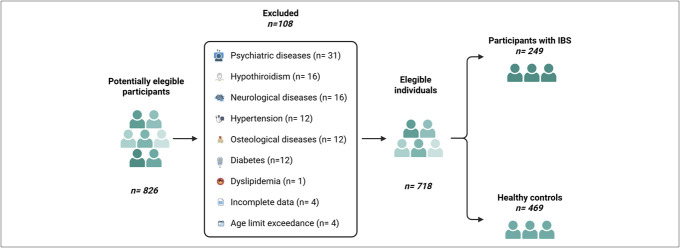
Flowchart showing the participant selection process. Individuals with comorbidities (including inflammatory and/or infectious colitis), those exceeding the age limit, and those with incomplete data were excluded.

The study initially enrolled 826 participants. After excluding individuals with comorbidities (n = 100), those exceeding the age limit (n = 4), and those with incomplete data (n = 4), the final cohort consisted of 718 individuals (60.7% women, median age 22, range: 18–73) (Figure 1). Among them, 469 were HCs (median age 22 interquartile range 20–26, 55.4% women) and 249 were subjects with IBS (median age 22 years, interquartile range 20–29, 70.7% women).

### IBS subtypes and severity

IBS subtypes were as follows: 123 subjects (49.4%) with constipation, 76 mixed (30.5%), 30 diarrhea (12%), and 20 unclassified (8%). Regarding IBS severity, 55.4% of subjects experienced mild IBS symptoms, 36.9% had moderate symptoms, and 7.6% had severe symptoms. Significant differences were observed between patients with IBS and healthy controls for anxiety, depression, and total IBS-SSS scores (Table 1). No significant differences were observed between married and nonmarried individuals with IBS.

**Table 1.** Comparison between individuals with IBS and healthy control

Variable	IBS (n = 249)	Control (n = 469)	*P* value
Age	24.8 (IQR 20–29)	26.3 (IQR 20–29)	0.039
Biological sex	Women 176 (70.7%)	Women 260 (55.4%)	<0.0001
	Men 73 (29.3%)	Men 209 (44.6%)	
BMI	24.34 [21.74–27.56]	24.46 [21.93–27.17]	0.864
Occupation	Student 158 (63.5%)	Student 357 (76.1%)	<0.0001
	Worker 73 (29.3%)	Worker 76 (16.2%)	
	Medical doctor 16 (6.4%)	Medical doctor 18 (3.8%)	
	Others 2 (0.8%)	Others 18 (3.8%)	
Education level	—	Elementary 1 (0.2%)	*0.022
		Jr Highschool 2 (0.4%)	
	Jr Highschool 2 (0.8%)	Highschool 186 (39.7%)	
	Highschool 81 (32.5%)	University 240 (51.2%)	
	University 126 (50.6%)	Postgrad 40	
	Postgrad 40 (16.1%)	8.5%	
Marital status	Single 208 (83.5%)	Single 423 (90.2%)	*0.009
	Married 41 (16.5%)	Married 46 (9.8%)	
HADS-A	10 [7–13]	7 [4–10]	<0.0001
HADS-D	7 [5–9]	6 [4–8]	<0.0001
IBS-SSS	161.6 [103.3–219.9]	66.6 [25–136.6]	<0.0001

The comparison between groups was conducted using the Student *t* test or Wilcoxon rank sum test as appropriate for numerical variables, or the χ^2^ test for categorical variables.

BMI, body mass index; HADS-A, Hospital Anxiety and Depression Scale, Anxiety subscale; HADS-D, Hospital Anxiety and Depression Scale, Depression subscale; IBS, Irritable Bowel Syndrome; IBS-SSS, Irritable Bowel Syndrome Severity Scale; IQR, interquartile range.

### Characteristics of the population by SOGI

**Figure 2.**
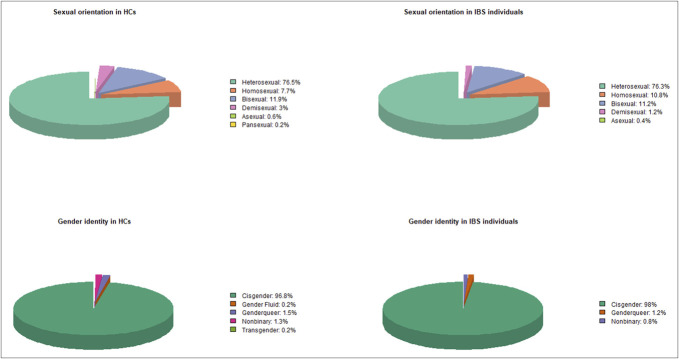
Graphic bars representing the SGM community in HCs and individuals with IBS. In HCs, 116 individuals (24.7%) identified as part of the SGM community. In the IBS group, 60 individuals (24.1%) identified as part of the SGM community. HC, healthy control; IBS, irritable bowel syndrome; SGM, sexual and gender minority.

Of the total of participants, 542 (75.5%) identified as cisgender heterosexuals, whereas 176 (24.5%) belonged to SGM communities. Regarding sexual orientation, 549 subjects (76.5%) identified as heterosexual, 84 (11.7%) as bisexual, 63 (8.8%) as gay/lesbian, 17 (2.4%) as demisexual, 4 (0.6%) as asexual, and 1 (0.1%) as pansexual. Regarding gender identity, 698 subjects (97.2%) identified as cisgender, 10 (1.4%) as genderqueer, 8 (1.1%) as nonbinary, 1 (0.1%) as gender-fluid, and 1 (0.1%) as transgender. SOGI characteristics among HCs and IBS individuals are detailed in Figure 2.

### Sexual orientation and symptoms severity among groups

When comparing the prevalence of sexual orientations between HCs and individuals with IBS, a similar proportion of heterosexuals (76.5% vs 76.3%, *P* = 0.942), gays (6.4% vs 7.6%, *P* = 0.624), bisexual men (3.6% vs 1.6%, *P* = 0.233), bisexual women (8.3% vs 9.6%, *P* = 0.684), and individuals identified with other orientations (3.8% vs 1.6%, *P* = 0.114) was observed. However, the prevalence of women who identified as lesbians was higher in IBS individuals compared with HCs (3.2% vs 1.3%, *P* = 0.041).

**Figure 3.**
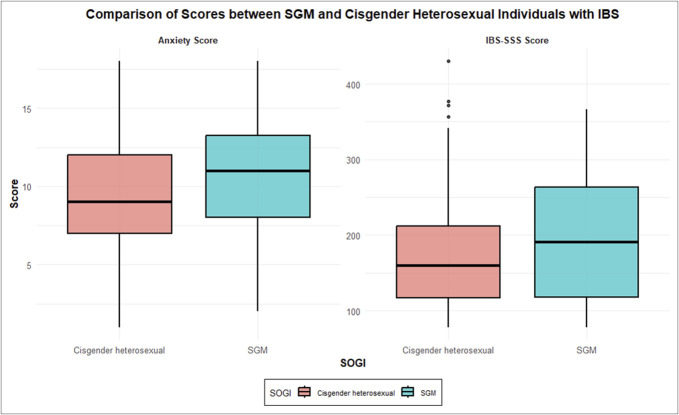
A box plot comparing scores on the HADS-A and IBS-SSS in cisgender heterosexual and SGM individuals with IBS is presented. The boxes represent LGBTQIA+ subjects (blue) and cisgender heterosexual subjects (pink). Distribution and homoscedasticity tests were evaluated. The Student *t* tests or Wilcoxon Rank-Sum tests were used for comparisons as appropriate. HADS-A, Hospital Anxiety and Depression Scale, anxiety subscale; IBS, irritable bowel syndrome; IBS-SSS, Irritable Bowel Syndrome Severity Symptom Scale; SGM, sexual and gender minority; LGBTQIA, lesbian, gay, bisexual, transgender, queer, intersex, asexual, and others.

Within the IBS group, SGM individuals reported higher IBS-SSS (190.75 [117.02–263.2] vs 159.9 [114.95–213.25], *P* = 0.032) and anxiety (11 [8–13.75] vs 9 [7–12], *P* = 0.032) when compared with cisgender heterosexual individuals (Table 2 and Figure 3). In addition, cisgender heterosexual participants were more likely to report mild IBS symptoms compared with SGM participants (59.5% vs 42.4%, *P* = 0.025; Table 3). Moreover, among lesbian women, there was a higher prevalence of unclassified IBS subtype compared with heterosexual women (30% vs 7.1%, *P* = 0.037).

**Table 2.** Comparison between SGM and cisgender heterosexual individuals with IBS

Variable	LGBTQIA+ with IBS (n = 60)	Cisgender heterosexuals with IBS (n = 189)	*P* value
Age	23 [range: 18–55]	22 [range: 18–62]	0.867
Biological sex	Woman 37 (61.7%)	Women 139 (73.5%)	0.103
	Men 23 (38.3%)	Men 50 (26.5%)	
BMI	24.66 [21.29–29.03]	24.34 [21.82–27.54]	0.437
Occupation	Student 35 (58.3%)	Student 123 (65.1%)	0.077
	Worker 17 (28.3%)	Worker 56 (29.6%)	
	Medical doctor 8 (13.3%)	Medical doctor 8 (4.2%)	
		Others 2 (1.1%)	
Education level	—	Jr Highschool 2 (1.1%)	0.734
	Highschool 21 (35%)	Highschool 60 (31.7%)	
	University 28 (46.7%)	University 98 (51.9%)	
	Postgrad 11 (18.3%)	Postgrad 29 (15.3%)	
Marital status	Singled 58 (96.7%)	Singled 150 (79.4%)	0.002
	Married 2 (3.3%)	Married 39 (20.6%)	
Have you ever visited a doctor for GI symptoms?	No 26 (43.3%)	No 92 (48.7%)	0.470
	Yes 34 (56.7%)	Yes 97 (51.3%)	
Perception of how much importance the physician attributed to their GI symptoms	Very important 11 (31.42%)	Very important 27 (25.47%)	0.759
	Somewhat important 15 (42.85%)	Somewhat important 51 (47.66%)	
	Not very important 9 (25.71%)	Not very important 26 (24.52%)	
		Not important at all 2 (1.88%)	
IBS Classification	Constipation 30 (50%)	Constipation 93 (49.2%)	0.312
	Mixed 15 (25%)	Mixed 61 (32.3%)	
	Diarrhea 7 (11.7%)	Diarrhea 23 (12.2%)	
	Unclassified 8 (13.3%)	Unclassified 12 (6.3%)	
HADS-A	11 [8–13.75]	9 [7–12]	*0.032
HADS-D	7 [5–9]	6 [4–9]	0.307
IBS-SSS score	190.75 [117.02–263.2]	159.9 [114.95–213.25]	*0.038

The comparison between groups was conducted using the Student *t* test or Wilcoxon rank sum test as appropriate for numerical variables, or the χ^2^ test for categorical variables.

BMI, body mass index; GI, gastrointestinal; HADS-A, Hospital Anxiety and Depression Scale, Anxiety subscale; HADS-D, Hospital Anxiety and Depression Scale, Depression subscale; IBS, Irritable Bowel Syndrome; IBS-SSS, Irritable Bowel Syndrome Severity Scale; SGM, sexual and gender minority.

**Table 3.** Comparison of scores and prevalence of sexual orientation and gender identity between IBS severity subgroups

Scores	Mild IBS (n = 138)	Moderate IBS (n = 92)	Severe IBS (n = 19)	*P* value
HADS-A	9 [6–12]	11 [8–13.75]	12 [8–14]	0.001
HADS-D	6 [4–8]	7 [6–10]	7 [4–8]	0.003
LGBTQIA+	26 (43.3%)	27 (45%)	7 (11.7%)	0.076
Cisgender heterosexual	112 (59.3%)	65(34.4%)	12 (6.3%)	
Heterosexual	113 (81.9%)	65 (70.7%)	12 (63.2%)	0.055
Gay	8 (5.8%)	9 (9.8%)	2 (10.5%)	0.453
Lesbian	3 (2.2%)	3 (3.3%)	2 (10.5%)	0.278
Bisexual men	2 (1.4%)	2 (2.2%)	—	0.771
Bisexual women	11 (8%)	10 (10.9%)	3 (15.8%)	0.490
Other	1 (0.7%)	3 (3.3%)	—	0.275

Multiple comparisons were conducted using the Kruskal-Wallis test. The χ^2^ test was used for categorical variables.

HADS-A, Hospital Anxiety and Depression Scale, Anxiety subscale; HADS-D, Hospital Anxiety and Depression Scale, Depression subscale; IBS, irritable bowel syndrome; LGBTQIA, lesbian, gay, bisexual, transgender, queer, intersex, asexual, and others.

When comparing the IBS-SSS total score among different sexual orientation groups, women identified as lesbian had a higher score, although it did not reach statistical significance.

In heterosexual-oriented cisgender individuals with IBS, age correlated with symptom impact on QOL (*r* = 0.165, *P* = 0.023) and inversely with anxiety (*r* = −0.151, *P* = 0.038). Anxiety also correlated with pain severity (*r* = 0.167, *P* = 0.021), number of days with pain (*r* = 0.259, *P* = 0.002), symptom impact on QOL (*r* = 0.238, *P* < 0.001), and the IBS-SSS total score (*r* = 0.219, *P* = 0.002). On the other hand, depression correlated with the number of days with pain (*r* = 0.253, *P* = 0.002), symptom impact on QOL (*r* = 0.223, *P* = 0.002), and the IBS-SSS total score (*r* = 0.163, *P* = 0.025).

In subjects with IBS belonging to the SGM communities, age correlated with distension severity (*r* = 0.348, *P* = 0.016), body mass index with pain severity (*r* = 0.325, *P* = 0.024), and depression score with the total IBS-SSS score (*r* = 0.290, *P* = 0.024).

In the logistic regression, the Nagelkerke pseudo-*R*^2^ was 0.087, and the Hosmer–Lemeshow showed an adequate fit to the data (*P* = 0.927). Anxiety (odds ratio [OR] 2.5, 95% CI, 1.8–3.5, *P* < 0.0001), depression (OR 1.4, 95% CI, 1.041–2.005, *P* = 0.027), biological sex (OR 1.9, 95%, 1.3–2.6, *P* < 0.0001) and being identified as lesbian (OR 2.7, 95% CI, 1–7.3, *P* = 0.04) were factors associated with IBS. In addition, being identified as a bisexual woman (OR 1.8, 95% CI, 1–3.6, *P* = 0.034) was a factor associated with anxiety.

## DISCUSSION

In this study, we compared the gastrointestinal symptom profiles of SGM and heterosexual individuals with IBS. SGM individuals with IBS reported significantly more severe gastrointestinal symptoms and higher levels of anxiety than their cisgender heterosexual counterparts. These findings highlight the critical influence of SOGI on the manifestation of IBS, suggesting that the unique stressors associated with being part of the SGM communities, may intensify both psychological distress and the severity of gastrointestinal symptoms.

It is noteworthy that the distribution of IBS subtypes in our sample aligns with previous reports in the literature. IBS-C and IBS-M were the most prevalent subtypes, accounting for 50–70% of cases, followed by IBS-D at 10–25%, with IBS-U being the least common. Importantly, the overall IBS prevalence of 35% observed in our study falls at the upper limit of what has been reported in systematic reviews (9–35%) (19). This relatively high prevalence may be partly explained by the characteristics of our sample, because most participants were affiliated with a university setting. This population may be subject to distinct psychosocial and environmental influences, including heightened stress levels, specific dietary habits, and greater health awareness, all of which could contribute to increased symptom recognition and reporting. These factors should be considered when assessing the generalizability of our findings.

Heteronormativity imposes traditional gender roles and sexual orientations, contributing to the marginalization and stigmatization of SGM individuals, particularly in healthcare settings. This marginalization fosters stigma, discrimination, and victimization, which complicate the accurate assessment and treatment of health disparities (20). The Minority Stress Model adapted for gastrointestinal diseases by Vélez et al (21) highlights the role of biopsychosocial factors and social determinants of health in shaping SGM health outcomes. SGM individuals are at greater risk of adverse life events, which are strongly associated with psychological distress, including higher rates of depression, anxiety, suicidal ideation, and chronic toxic stress, factors that disrupt the microbiota-gut-brain axis and contribute to gastrointestinal disorders, increased morbidity, and mortality (21,22). Furthermore, social determinants such as diet, socioeconomic status, and limited access to specialized care exacerbate these disparities (23). To reduce these inequities and improve quality of life, it is essential for healthcare policies and societal norms to evolve toward greater inclusivity and support for the mental, emotional, and physical well-being of SGM communities (24–27).

Gender identity and sexual orientation are intricate and complex traits that are believed to develop during early developmental stages (28). Understanding these aspects is crucial for public health, because they significantly influence individuals' well-being and healthcare needs (28,29). Recent studies show that over 20% of Generation Z identifies as being of SGM gender identity (21), closely aligned with the 24.5% prevalence observed in our study, which predominantly involved participants with a median age of 22, due to the Research Institute association to a university. This finding underscores the increasing imperative to train healthcare professionals to provide inclusive and cultural humble carefree of implicit bias. Recognizing the complexity of these traits helps to develop more effective and comprehensive health policies and ensures that interventions are sensitive to the unique challenges SGM populations face. This includes being aware of specific issues (cultural bias, gender-affirming hormone treatment, minority stress, etc.) related to the SGM population to ensure that their unique health needs are met effectively. Such understanding is essential for fostering environments that support the health and dignity of all individuals, regardless of their gender identity or sexual orientation.

Research into the health disparities experienced by SGM populations highlights a multifaceted interplay of neurodevelopmental, psychiatric, behavioral, pharmacologic, anatomic, and psychosocial stressors (22). These studies consistently demonstrate that individuals identifying as SGM are more susceptible to conditions such as attention deficit hyperactivity disorder, bipolar disorder, eating disorders (30), sleep disturbances (31,32), depression, anxiety, autism, obesity, and chronic pain (33,34). Contributing factors include prenatal influences on brain development, which predispose individuals to these conditions and influence gender behavioral tendencies (35). Notably, these factors have also been linked to DGBI (36–39).

In Mexico and globally, SGM individuals face systemic challenges that exacerbate their mental health risks. Inadequate institutional protections and conservative cultural attitudes toward gender and sexuality intensify these. There is a significant deficiency in inclusive and culturally humble training and targeted SGM awareness among healthcare professionals, leading to suboptimal care and services. Although we hypothesized that various SGM populations, due to complex multisystem factors, may be at a higher risk of developing IBS and experiencing worse health-related outcomes, the similar proportion of CIS and SGM individuals (except for lesbian women) among both HCs and IBS subjects suggests that the perception of illness, particularly the severity of symptoms, rather than the mere presence of IBS, may be a key differentiating factor between these groups.

Our research has elucidated that among SGM communities, certain subgroups, notably lesbians, exhibit distinctive patterns of IBS symptoms. These patterns could be influenced by specific psychosocial stressors that remain inadequately understood. Some studies indicate that lesbian and bisexual women (sexual minority women) experience a disproportionately high prevalence of health disparities such as increased rates of smoking, alcohol consumption, back pain, headache/migraine, obesity, asthma, certain types of cancer, and disability, coupled with poorer overall health (40–43), Moreover, these women are more susceptible to frequent mental distress compared with their heterosexual counterparts (44). It is a well-documented fact that gastrointestinal symptoms and psychological distress are more prevalent in women than in men. In addition, individuals within the SGM communities face unique stressors related to their SOGI. These factors likely contribute to the observed increased prevalence, and unique symptomatology of IBS in these subgroups, suggesting a complex interplay of biological and socioenvironmental factors that warrants further investigation.

According to our knowledge, this is the first study worldwide to compare the clinical profile of heterosexual individuals with IBS and SGM individuals; however, our study has some limitations to be acknowledged. The SGM representation within our sample may not adequately reflect the extensive diversity inherent to the broader SGM communities, thus constraining the generalizability of our findings. In addition, the reliance on self-reported data raises the potential for response bias. Our recruitment strategy, which primarily used electronic surveys, may not have captured a representative cross-section of the broader Mexican population, introducing a risk of selection bias. A key limitation of the study is the overrepresentation of individuals affiliated with a university campus, leading to a sample with higher educational levels and distinct socioeconomic characteristics compared with the general Mexican population. This may introduce bias and limit the external validity of the findings, because the results may not be generalizable to populations with different educational or sociocultural backgrounds. In addition, factors such as genetics, dietary habits, and physical activity, which were not controlled for, could also influence the outcomes. Notably, the median age of our participants was 22 years, which might limit the applicability of our results to older populations. Moreover, anxiety may be reflective of symptom specific anxiety (45,46), versus a *bona fide* anxiety-spectrum disorder.

Further research is crucial to explore the individual experiences among SGM individuals with IBS, identify specific stressors, and elucidate the biological mechanisms underlying these associations. It is essential to account for cultural differences, as norms and values can shape experiences of discrimination, stress, and coping mechanisms. Given the sociocultural specificity of our sample, it is essential that these findings be replicated in diverse contexts. Cultural environments play a significant role in shaping both the lived experiences of SGM (sexual and gender minority) individuals and the expression and perception of gastrointestinal symptoms. In countries with more inclusive social climates and equitable healthcare systems, different patterns may emerge, driven by reduced exposure to minority stress, improved access to affirming care, and broader societal acceptance. Conversely, in settings marked by structural stigma or limited healthcare access, symptom severity and reporting may be exacerbated.

This variability underscores the importance of interpreting findings within the local sociopolitical and healthcare context and highlights the need for cross-cultural research to better understand the interplay between identity, psychosocial stressors, and digestive health. In addition, longitudinal studies are needed to examine how these symptoms evolve over time and how they are influenced by personal, contextual, and structural changes. Such an approach would offer deeper insight into the underlying mechanisms and support the development of more effective, tailored interventions. Future research should also continue to collect detailed SOGI data to enhance understanding and improve health outcomes within these communities.

SGM individuals with IBS experienced more severe symptoms and greater anxiety than their cisgender heterosexual counterparts. The observed disparities in IBS severity and psychological distress between SGM and cisgender heterosexual-oriented individuals highlight the importance of considering SOGI in health care. Addressing these differences could lead to more effective and inclusive management strategies for IBS, ensuring that the unique needs of all individuals are recognized and appropriately treated.

## CONFLICTS OF INTEREST

**Guarantor of the article:** José María Remes-Troche, MD.

**Specific author contributions:** S.A.R.-D., collecting and interpreting data, and drafting the manuscript; B.A.P.-P., planning the study, collecting and interpreting data, and drafting the manuscript; H.R.O.-A., collecting and interpreting data, E.L.N.-J., collecting and interpreting data; C.L.D.-N., collecting and interpreting data; F.H.-d.l.T., collecting and interpreting data; M.A.-B., reviewing the work critically for important intellectual content; C.V., drafting the work and reviewing it critically for important intellectual content; J.M.R.-T., drafting the work and reviewing it critically for important intellectual content. All authors have approved the final draft submitted.

**Financial support:** None to report.

**Potential competing interests:** None to report.

**Study Highlights**

**WHAT IS KNOWN**

✓ Sexual and gender minority individuals with irritable bowel syndrome (IBS) experience more severe gastrointestinal symptoms than cisgender heterosexual counterparts.
✓ Mental health disorders, such as anxiety, often accompany gastrointestinal symptoms in individuals with IBS.

**WHAT IS NEW HERE**

✓ This is the first study in Mexico to explore the intersection of IBS, sexual orientation, and gender identity.
✓ Sexual and gender minority participants with IBS reported significantly higher levels of anxiety than cisgender heterosexuals.
✓ These findings underscore the need for further inclusive research in other countries to determine whether similar patterns are present.

## ABBREVIATIONS:

BMI: body mass index
CI: confidence interval
DGBI: disorder of gut-brain interaction
HAD: Hospital Anxiety and Depression Scale
HC: health control
IBS: irritable bowel syndrome
IQR: interquartile range
ISS-SSS: Irritable Bowel Syndrome Severity Scale
LGBTQIA+: lesbian, gay, bisexual, transgender, queer, intersex, asexual, and others
OR: odds ratio
QOL: quality of life
SGM: sexual and gender minority
SOGI: sexual orientation and gender identity
WHO: World Health Organization
